# Soluble immune checkpoint molecules in patients with antineutrophil cytoplasmic antibody-associated vasculitis

**DOI:** 10.1038/s41598-022-25466-x

**Published:** 2022-12-09

**Authors:** Jung Yoon Pyo, Taejun Yoon, Sung Soo Ahn, Jason Jungsik Song, Yong-Beom Park, Sang-Won Lee

**Affiliations:** 1 Division of Rheumatology, Department of Internal Medicine, Yonsei University College of Medicine, 50-1 Yonsei-Ro, Seodaemun–gu, Seoul, 03722 Republic of Korea; 2 Department of Medical Science, BK21 Plus Project, College of Medicine, Yonsei University, Seoul, Republic of Korea; 3 Institute for Immunology and Immunological Diseases, Yonsei University College of Medicine, Seoul, Republic of Korea

**Keywords:** Autoimmunity, Rheumatology, Rheumatic diseases

## Abstract

Immune checkpoint molecules balance immune effector responses with regulatory reactions. We speculated that soluble immune checkpoint molecules are involved in dysregulation of the immune response and autoimmunity. We evaluated the association between soluble immune checkpoint molecules and antineutrophil cytoplasmic antibody (ANCA)-associated vasculitis (AAV). A total of 56 patients with AAV from a prospective observational cohort and 40 healthy controls (HCs) were analyzed. Soluble PD-1, PD-L1, PD-L2, CTLA-4, CD28, CD80, CD86, ICOS, TIM-3, BTLA, CD40, LAG-3, TLR-2, and CD27 were measured in stored sera using the Milliplex MAP assay. Paired analyses were performed before and after the treatment. AAV-specific indices, including Birmingham vasculitis activity score, five factor score , vasculitis damage index, and blood samples, were collected. Patients with AAV had higher levels of sPD-L1, sCD28, sCD80, sCD86, sICOS, sTIM-3, sLAG-3, sTLR-2, and sCD27 and lower level of sCTLA-4 than HCs (*p* < 0.05). Patients with AAV had higher serum sCD28, sCD80, sTIM-3, and sCD27 levels than HCs at baseline and decreased after treatment. Furthermore, the serum levels of sCD28 and sTIM-3 were significantly correlated with disease activity. This study demonstrated altered concentrations of serum soluble immune checkpoint molecules in patients with AAV. In particular, sCD28 and sTIM-3 may act as surrogate markers of AAV disease activity.

## Introduction

Antineutrophil cytoplasmic antibody (ANCA)-associated vasculitis (AAV) is a systemic vasculitis involving small vessels and includes microscopic polyangiitis (MPA), granulomatosis with polyangiitis (GPA), and eosinophilic GPA (EGPA). AAV is a systemic autoimmune disease, and the presence of ANCA is a hallmark of the disease. Binding ANCA to neutrophil myeloperoxidase (MPO) or proteinase 3 (PR3) results in neutrophil activation, which causes endothelial injury and inflammation^1^.

Immune checkpoint molecules are co-stimulatory or co-inhibitory receptors and ligands that balance immune effectors and regulatory reactions^2^. Autoimmunity is prevented by immune tolerance, and immune checkpoint molecules play a decisive role in maintaining homeostasis between immune stimulation and inhibition^3,4^. Dysregulation of immune homeostasis may lead to a break of immune tolerance and develop autoimmunity^5^.

Immune checkpoint molecules are expressed on the cell surface and function by interacting with their ligands. Soluble forms of these immune checkpoint molecules are produced by proteolytic cleavage of the surface molecule or alternative splicing of mRNA^6,7^. Previous studies have shown aberrant expression of soluble checkpoint molecules in various autoimmune diseases, such as systemic lupus erythematosus (SLE)^8^, rheumatoid arthritis (RA)^9–11^, and autoimmune thyroid diseases^12^.

Based on these findings, we hypothesized that soluble immune checkpoint molecules are involved in immune dysregulation by interfering with the signaling between surface immune checkpoint molecules. However, little is known about the functions of soluble immune checkpoint molecules in AAV and their correlation with AAV disease activity. Here, as an attempt to further elucidate the immunopathological roles of soluble immune checkpoint molecules and to investigate for potential surrogate markers in AAV, we analysed the serum concentration and ex vivo production of various soluble immune checkpoint molecules from peripheral blood monomuclear cells (PBMC) in patients with active and inactive AAV and compared them with healthy controls. Novel discoveries regarding soluble immune checkpoint molecules may provide insights into the pathophysiological mechanisms underlying AAV.

## Patients and Methods

### Patients

Fifty-six patients with AAV and 40 healthy controls (HCs) were included in this study. All patients were enrolled in the Severance Hospital ANCA-associated VasculitidEs (SHAVE) cohort, a prospective observational cohort of patients with MPA, GPA, and EGPA established in November 2016. AAV diagnosis was made according to the 2007 European Medicine Agency algorithms for AAV and polyarteritis nodosa and the 2012 revised International Chapel Hill Consensus Conference Nomenclature of Vasculitides^13,14^. At the time of diagnosis, i) patients who received immunosuppressive drugs, ii) those who had a follow-up period of < 3 months, and iii) those presenting with serious concomitant medical conditions, such as malignancy, serious infection, and other systemic vasculitides other than AAV, were excluded. This study was approved by the Institutional Review Board (IRB) of Severance Hospital (4–2016-0901), and written informed consent was obtained from the patients at the time of blood sampling.

### Clinical and laboratory data

Demographic data, including age and sex, were collected. The AAV subtypes and clinical manifestations were evaluated based on the nine categories of the Birmingham Vasculitis Activity Score (BVAS). MPO-ANCA, proteinase 3 (PR3)-ANCA, acute-phase reactants, erythrocyte sedimentation rate (ESR), and C-reactive protein (CRP) were measured. We collected AAV-specific indices, BVAS version 3 for disease activity^15^, five-factor score (FFS) for predicting prognosis^16^, and vasculitis damage index (VDI) for assessment of damage^17^ on the same day of blood sampling.

### Blood collection and storage

Paired blood samples were collected from consecutive patients with active and inactive AAV in the same patient. Whole blood was collected from the patients, PBMCs were isolated by Ficoll density-gradient centrifugation, and sera were immediately stored at –80 °C. Active AAV was defined as the highest BVAS state during the follow-up period, and inactive AAV was defined as the lowest BVAS state after treatment with active AAV. Of the patients with active AAV, 41 (73.2%) were sampled for newly diagnosed disease and 15 (26.8%) for relapsed disease. All patients in the inactive state of AAV were available for the second sample.

### Measurement of soluble immune checkpoint molecules

We measured the serum concentrations of soluble checkpoint molecules in patients with AAV and in HCs using a custom-made multiplex assay (Milliplex, Merck, Darmstadt, Germany). This multiplex assay kit includes 17 checkpoint molecules: programmed cell death (PD)-1, programmed cell death-ligand1 (PD-L1), PD-L2, cytotoxic T-lymphocyte antigen-4 (CTLA-4), cluster of differentiation (CD)28, CD80, CD86, inducible T-cell co-stimulator (ICOS), T-cell immunoglobulin and mucin-domain containing-3 (TIM-3), herpes virus entry mediator (HVEM), B- and T-lymphocyte attenuator (BTLA), CD40, lymphocyte-activation gene 3 (LAG-3), toll-like receptor 2 (TLR-2), CD27, glucocorticoid-induced TNFR-related protein (GITR), and GITRL. A Luminex 200 Bio-Plex instrument (Bio-Rad, CA, USA) was used to analyze the concentrations of these molecules, according to the manufacturer’s instructions. HVEM, GITR, and GITRL were excluded from the analysis because more than 70% of the data were missing. The cutoff values for each molecule were set at the mean plus two-fold the standard deviation (SD) of 40 HCs.

### Flow cytometry

We obtained PBMCs from 10 patients with AAV with a high BVAS (BVAS > 15) and 10 patients with AAV with a low BVAS (BVAS < 5) and analyzed the surface expression of PD-1, Tim-3, and CD28 on T cells using flow cytometry. The following antibodies were used for staining: anti-CD3-V500 (BD Biosciences, Oxford, UK), anti-CD4-Alexa Fluor 700, anti-CD25-APC, anti-CD28-PE-Cy7, anti-CD279 (PD-1)-BV421, and anti-CD388 (Tim-3)-PE ( BioLegend, CA, USA). Lymphocytes were gated using forward and side scatter parameters, and samples were analyzed using FACSVerse (BD Biosciences, Oxford, UK) and associated software programs (FlowJo).

### HCs

Serum levels of soluble immune checkpoint molecules were measured in the sera of 40 HCs who underwent medical check-ups. The use of clinical data from HCs was approved by the IRB of Severance Hospital (4–2017-0761).

### Statistical analyses

All statistical analyses were performed using SPSS software ver. 26 (IBM Corp., Armonk, NY, USA). Categorical data are expressed as absolute and relative frequencies, and continuous data are presented as median (quartile range). The Mann–Whitney U test was performed to compare continuous variables, whereas the chi-square or Fisher’s exact test was used to analyze categorical variables. The correlation coefficient was obtained using Pearson’s correlation analysis or Spearman’s correlation analysis for non-normally distributed data. The Wilcoxon signed-rank test was used to analyze paired data. Statistical significance was set at *p* < 0.05.

### Ethics

This study was approved by the Institutional Review Board (IRB) of Severance Hospital (4-2016-0901) and was conducted in accordance with the Declaration of Helsinki. Written informed consent was obtained from the patients at the time of enrolment in the SHAVE cohort and blood sampling.

## Results

### Baseline demographics

The baseline demographic characteristics of the 56 patients included in this study are summarized in Table 1. The median age was 64.5 and 65.0 years in the active and inactive states, respectively, and 20 patients were men. At the time of enrollment, 41 (73.2%) patients were newly diagnosed and 15 (26.8%) patients were relapsed. The median BVAS, FFS, and VDI scores were 12.0, 0.5, and 3.0 in patients with active AAV and 4.0, 0.0, and 3.0 in those with inactive AAV, respectively. The median time interval between the active and inactive states was 13.5 months. The AAV-specific indices and clinical manifestations in patients with active and inactive AAV are shown in Table 1.

**Table 1:** Characteristics of 56 patients with active and inactive AAV (Paired analyses).

Variables at the time of blood collection	Active state	Inactive state	*P* value
**Demographic data**			
Age (years)	64.5 (60.0)	65.0 (62.0)	< 0.001
Male sex (N, (%))	20 (35.7)	Same as left	N/A
Newly diagnosed disease	41 (73.2)	–	
Relapsing disease	15 (26.8)	–	
Disease duration (month)	0.0 (5.0)	13.5 (11.0)	N/A
**AAV subtypes (N, (%))**			
MPA	29 (51.8)	Same as left	N/A
GPA	15 (26.8)	Same as left	N/A
EGPA	12 (21.4)	Same as left	N/A
**ANCA positivity (N, (%))**			
MPO-ANCA(or P-ANCA) positivity	38 (67.9)	23 (41.1)	0.002
PR3-ANCA (or C-ANCA) positivity	6 (10.7)	6 (10.7)	0.172
Both ANCAs	1 (1.8)	0 (0.0)	–
ANCA negativity	11 (19.6)	26 (46.4)	0.001
No results	2 (3.6)	1 (1.8)	–
**AAV-specific indices**			
BVAS	12.0 (12.0)	4.0 (2.0)	< 0.001
FFS	0.5 (2.0)	0.0 (1.0)	< 0.001
VDI	3.0 (2.0)	3.0 (3.0)	0.038
**Clinical manifestations (N, (%))**			
General	26 (46.4)	2 (3.6)	< 0.001
Cutaneous	10 (17.9)	6 (10.7)	0.280
Mucous/Eye	2 (3.6)	3 (5.4)	1.000
Otorhinolaryngologic	23 (41.1)	20 (35.7)	0.560
Pulmonary	41 (73.2)	31 (55.4)	0.049
Cardiovascular	4 (7.0)	1 (1.8)	0.206
Gastrointestinal	5 (8.9)	0 (0.0)	0.495
Renal	33 (58.9)	28 (50.0)	0.343
Nervous	18 (32.1)	16 (28.6)	0.681
**Acute phase reactants**			
ESR (mm/hr)	35.0 (76.0)	15.0 (18.0)	0.001
CRP (mg/L)	5.4 (51.9)	1.5 (4.0)	0.004
**Until blood collection**			
*Immunosuppressive drugs administered (N, (%))*			
Glucocorticoids	14 (25.0)	55 (98.2)	
Cyclophosphamide	10 (17.6)	36 (64.3)	
Rituximab	2 (3.6)	12 (21.4)	
Azathioprine	6 (10.7)	43 (76.8)	
Mycophenolate mofetil	1 (1.8)	14 (25.0)	
Tacrolimus	0 (0.0)	5 (8.9)	
Methotrexate	2 (3.6)	6 (10.7)	

Values are expressed as median (interquartile range (IQR)) or number (percentage).

*AAV* ANCA-associated vasculitis, *ANCA* Antineutrophil cytoplasmic antibody, *MPA* Microscopic polyangiitis, *GPA* Granulomatosis with polyangiitis, *EGPA* Eosinophilic granulomatosis with polyangiitis, *MPO* Myeloperoxidase, P Perinuclear, *PR3* Proteinase 3, *C* Cytoplasmic, *SF-36* Short-form 36-item, *PCS* Physical component summary, *MCS* Mental component summary, *BVAS* Birmingham vasculitis activity score, *FFS* Five-factor score, *VDI* Vasculitis damage index, *ESR* Erythrocyte sedimentation rate, *CRP* C-reactive protein, *N/A* Not applicable.

### Serum concentrations of the soluble immune checkpoint molecules and AAV-specific indices

**Figure 1:**
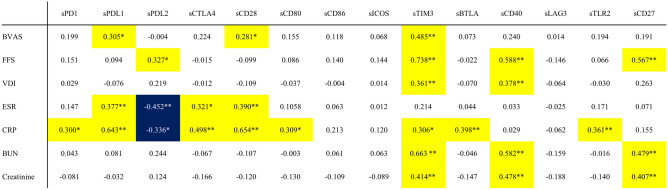
Pearson correlation analysis between serum soluble checkpoint molecule concentrations and disease activity markers of AAV in patients with active AAV. Correlations between disease activity markers and the concentrations of soluble checkpoint molecules are shown. Yellow: positive correlation; blue: negative correlation. * *P *< 0.05, ** *P *< 0.01. *sPD* Soluble programmed cell death, *sPD-L* Soluble programmed cell death ligand, *sCTLA-4* Soluble cytotoxic T-lymphocyte antigen 4, *sCD* Soluble cluster of differentiation, *sICOS* Soluble inducible T-cell costimulatory, *sTIM-3* Soluble T-cell immunoglobulin and mucin-domain containing-3, *sBTLA* Soluble B- and T-lymphocyte attenuator, *sLAG-3* Soluble lymphocyte-activation gene 3, *sTLR-2* Soluble toll-like receptor 2, *BVAS* Birmingham vasculitis activity score, *FFS* Five-factor score, *VDI* Vasculitis damage index, *ESR* Erythrocyte sedimentation rate, *CRP* C-reactive protein, *BUN* Blood urea nitrogen.

The correlations between disease activity markers, including AAV-specific indices and soluble immune checkpoint molecules, are shown in Fig. 1. Regarding disease activity, sPD-L1 (r = 0.305, *P *= 0.022), sCD28 (r = 0.281, *P *= 0.036), and sTIM-3 (r = 0.485, *P *< 0.001) were positively correlated with BVAS. Furthermore, these three molecules demonstrated positive correlations with acute-phase reactants. sPD-L1 correlated with ESR (r = 0.377, *P *= 0.005) and CRP (r = 0.643, *P *< 0.001), sCD28 was correlated with ESR (r = 0.390, *P *= 0.004) and CRP (r = 0.654, *P *< 0.001), and sTIM-3 was correlated with CRP (r = 0.306, *P *= 0.023). In contrast, sPD-L2 was negatively correlated with ESR (r =  − 0.452, *P *= 0.001) and CRP (r =  − 0.336, *P *= 0.012).

Furthermore, sPD-L2 (r = 0.327, *P *= 0.014), sTIM-3 (r = 0.738, *P *< 0.001), sCD40 (r = 0.588, *P *< 0.001), and sCD27 (r = 0.567, *P *< 0.001) were positively correlated with FFS. Regarding the severity of damage, sTIM-3 (r = 0.361, *P *= 0.006) and sCD40 (r = 0.378, *P *= 0.004) showed a positive correlation with VDI.

Regarding the relation between the soluble immune checkpoint molecules and kidney function, sTIM-3 (r = 0.663, *P *< 0.001 ; r = 0.414, *P *= 0.002), sCD40 (r = 0.582, *P *< 0.001 ; r = 0.478, *P *< 0.001), and sCD27 3 (r = 0.479, *P *< 0.001 ; r = 0.407, *P *= 0.002) demonstrated positive correlations with serum blood urea nitrogen (BUN) and creatinine, respectively.

### Serum concentrations of the soluble immune checkpoint molecules in patients with AAV and HCs

**Figure 2:**
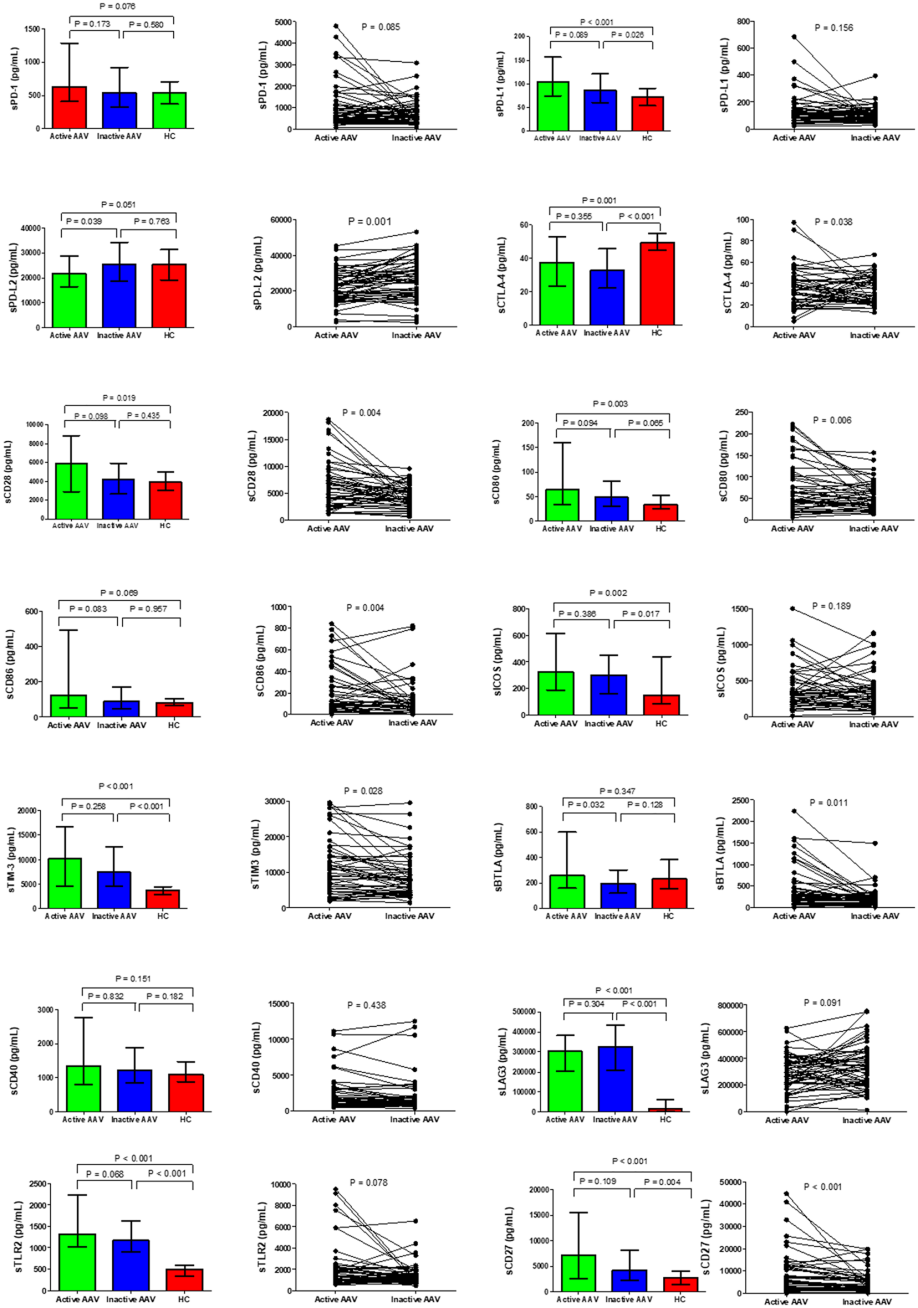
Serum concentrations of soluble checkpoint inhibitors in patients with active AAV, inactive AAV (after treatment), and healthy controls (HCs). Left side: median serum concentration with interquantile rage, Mann–Whitney U test applied for unpaired tests; Right side: paired samples for the 56 same patients, Wilcoxon signed-rank test applied. Plots show median, with error bars indicating ± interquartile range. *sPD* Soluble programmed cell death, *sPD-L* Soluble programmed cell death ligand, *sCTLA-4* Soluble cytotoxic T-lymphocyte antigen 4, *sCD* Soluble cluster of differentiation, *sICOS* Soluble inducible T-cell costimulatory, *sTIM-3* Soluble T-cell immunoglobulin and mucin-domain containing-3, *sBTLA* Soluble B- and T-lymphocyte attenuator, *sLAG-3* Soluble lymphocyte-activation gene 3, *sTLR-2* Soluble toll like receptor 2.

Patients with AAV had significantly higher serum concentrations of sPD-L1, sCD28, sCD80, sICOS, sTIM-3, sLAG-3, sTLR-2, and sCD27 than HCs, and the serum concentrations of sCTLA-4 were lower in patients with AAV than those in HCs (Fig. 2).

### Effect of activity of AAV on the production of soluble immune checkpoint molecules

We evaluated longitudinal changes in soluble immune checkpoint molecules from the active AAV state to the inactive AAV state after treatment. The serum concentrations of sCTLA-4, sCD28, sCD80, sCD86, sTIM-3, sBTLA, and sCD27 significantly decreased in parallel with BVAS. In contrast, the sPD-L2 levels significantly increased after treatment (Fig. 2).

### Expression of PD-1, CD28, and Tim-3 on CD4 + T cells

Among the soluble immune checkpoint molecules that decreased when disease activity changed from active state to inactive state, we considered that the molecules showing a positive correlation with BVAS were significant, which were sPD-L1, sCD28 and sTIM-3. Since we tested the surface expression of T-cells, we measured PD-1, which is the receptor of PD-L1.

**Figure 3:**
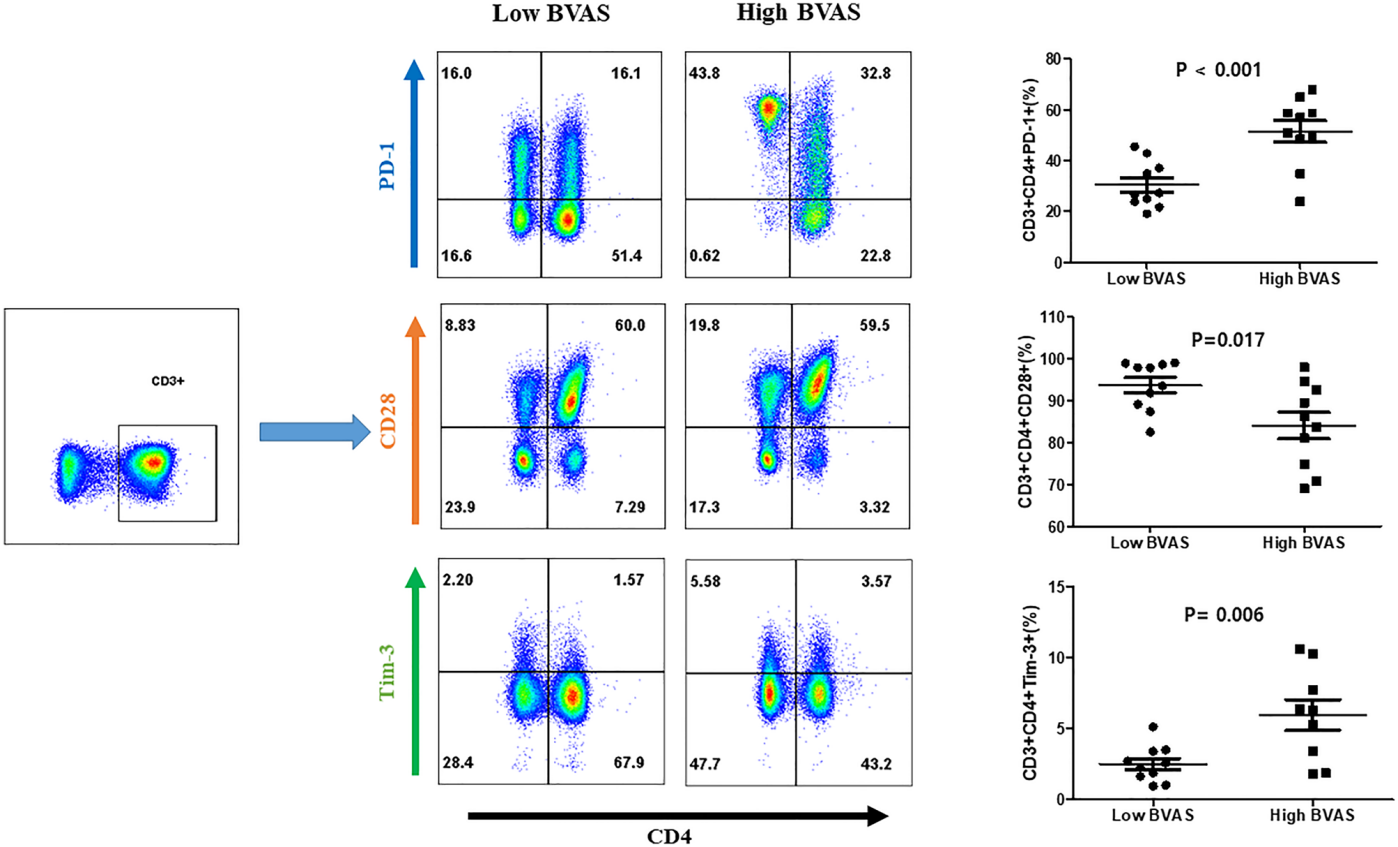
Comparison of the population of T cells expressing CD28 between patients with AAV with low BVAS and those with high BVAS. Patients with AAV with a high BVAS showed a significantly higher population of CD3 + CD4 + PD1 + T cells as well as CD3 + CD4 + Tim3 + T cells and a lower population of CD3 + CD4 + CD28 + T cells than those with a low BVAS. Plots show median, with error bars indicating ± interquartile range. *AAV* ANCA-associated vasculitis, *ANCA* Antineutrophil cytoplasmic antibody, *BVAS* Birmingham vasculitis activity score, *PD* Programmed cell death, *Tim-3* T-cell immunoglobulin and mucin-domain containing-3.

We collected the PBMCs from 10 patients with AAV with a high BVAS (BVAS > 15) and 10 patients with AAV with a low BVAS (BVAS < 5) to compare the cell surface expression of PD-1, CD28, and Tim-3 on CD4 + T cells. The population of PD-1 expressing CD4^+^ T cells was significantly higher in patients with high BVAS AAV than in those with low BVAS AAV (53.9% vs. 27.0%; *p *< 0.001). The population of Tim-3 expressing CD4^+^ T cells was significantly higher in patients with high BVAS AAV than in those with low BVAS AAV (6.35% vs. 2.42%; *p *= 0.006). Conversly, the population of CD28 expression in CD4^+^ T cells was higher in patients with low BVAS than in those with high BVAS (85% vs. 95.65%; *p *= 0.017) (Fig. 3).

## Discussion

Previous studies have demonstrated altered cell surface expression of checkpoint molecules in various autoimmune diseases^18–20^. Furthermore, it was considered that their aberrant expression could switch off immune tolerance, leading to the initiation of autoimmunity^4,21^. However, there has been no study clarifying the levels of the production of soluble forms of immune checkpoint molecules and determining their clinical roles in AAV patients to date.

This study demonstrated that the aberrant production of soluble checkpoint molecules might be associated with AAV. To the best of our knowledge, this is the first study to elucidate the association of the soluble immune checkpoint molecules with AAV-specific indices and acute phase reactants. Our results showed that sPD-L1, sCD28, sCD80, sICOS, sTIM-3, sLAG-3, sTLR-2, and sCD27 were significantly higher and sCTLA-4 was significantly lower in patients with AAV than those in HCs. The presence and altered production of these soluble checkpoint molecules may indicate that they play an important role in the derangement of immune homeostasis.

It is noteworthy that patients with active AAV had higher concentrations of sCD28 and sTIM-3 than HCs, which decreased as the disease improved after treatment. Furthermore, these soluble molecules were positively correlated with BVAS and CRP levels, indicating that sCD28 and sTIM-3 reflect disease activity. These findings are consistent with those of previous studies, which reported that plasma sCD28 and sTIM-3 concentrations were increased in patients with SLE and correlated with disease activity^22–24^. Additionally, a previous study showed that sCD28 concentrations were elevated in patients with RA and decreased after treatment^9^. The above observations and our results suggest that sCD28 and sTIM-3 could potentially serve as a surrogate biomarker of AAV disease activity. Based on these findings, we expected that soluble immune checkpoint molecules would be clinically useful if they could predict disease relapse or treatment failure. Additional prospective studies with serial measurements of the soluble checkpoint molecules would provide more reliable information regarding the clinical implications of using the soluble checkpoint molecules as a disease activity indicator for AAV patients.

Soluble immune checkpoint molecules are produced by proteolytic cleavage of the surface molecule or alternative splicing of mRNA [6.7]. There may be a possibility that the membrane-bound receptors may be released to the blood circulation from cells through apoptosis or necrosis; however, since their molecular weights are relatively heavy, and thus those receptors are generally precipitated by centrifugation, this possibility could be ignored.

CD28 is proteolytically cleaved in activated T cells by matrix metalloproteinases (MMP) 2 and MMP 13^25^, resulting in increased serum sCD28 levels. In our data, sCD28 levels were higher in patients with active AAV than in those with inactive AAV or HCs, and the surface expression of CD28 in CD4^+^ T cells was lower in patients with high BVAS. We speculated that the shedding of surface CD28 in its soluble form acts as a regulatory mechanism that inhibits the CD27:B7 activation pathway.

**Figure 4:**
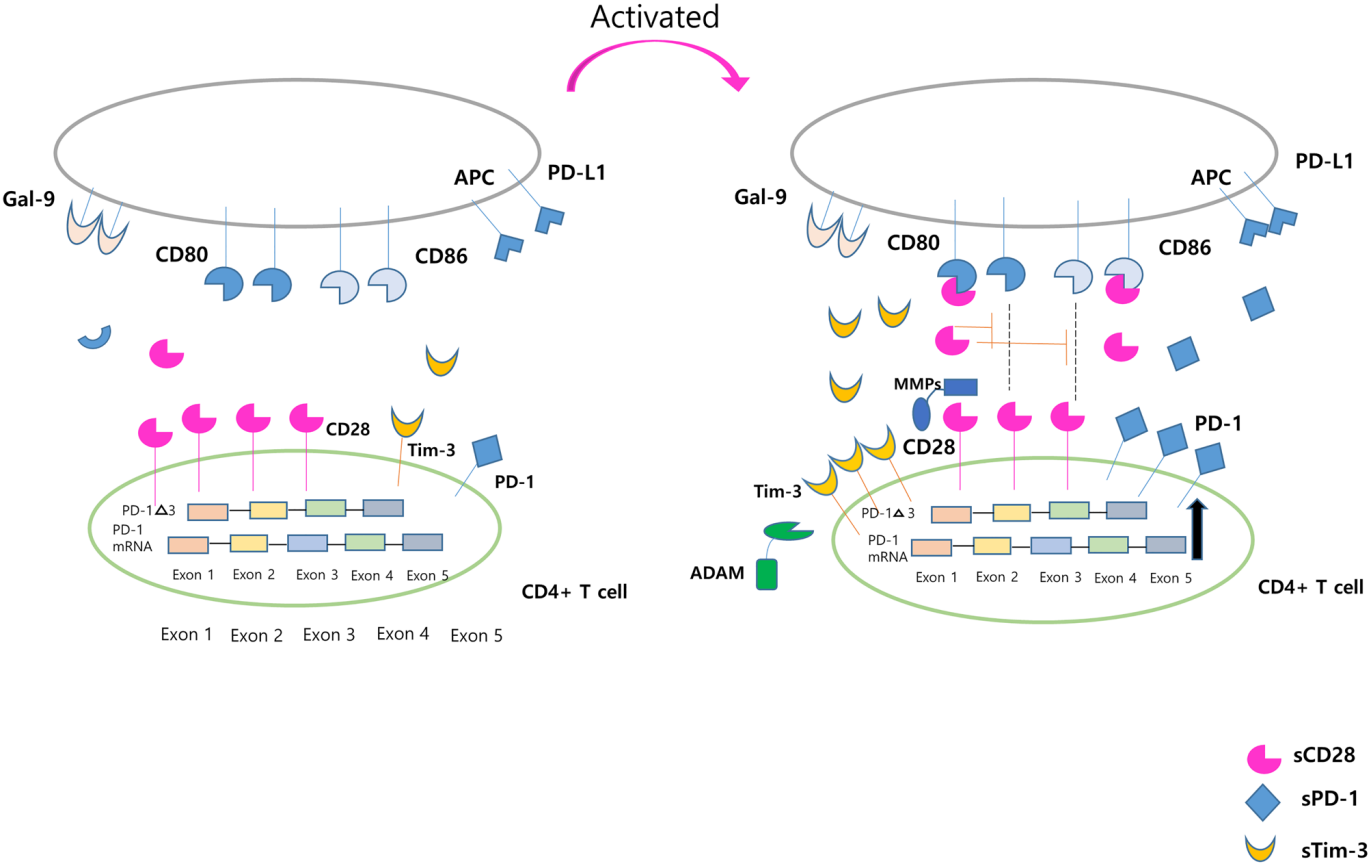
Schematic illustration of checkpoint molecule alteration in T cells by AAV activation. CD28 is proteolytically cleaved by MMP2 and MMP13 upon T-cell activation, resulting in the increased serum concentrations of sCD28 and decreased surface CD28 expression, and sCD28 acts as a decoy receptor. However, the production of inhibitory molecules, such as PD-1 and Tim-3, is induced by the persistent stimulation of T cell receptors. Consequentially, the serum concentration of sPD-1 produced by alternative splicing increases, and the serum levels of sTim-3 are increased by shedding by ADAM10 and 17. *AAV* ANCA-associated vasculitis, *ANCA* Antineutrophil cytoplasmic antibody, *MMP* Matrix metalloproteinases, *PD* Programmed cell death, *Tim-3* T-cell immunoglobulin and mucin-domain containing-3, *ADAM* A disintegrin and metalloproteinase domain-containing protein, *Gal-9 (Tim-3 ligand)* Galectin-9, *PD-L1* Programmed cell death ligand-1.

The surface expression of PD-1 and Tim-3 on CD4^+^ T cells was higher in patients with a high BVAS than in those with a low BVAS in our data. PD-1 and Tim-3 are representative inhibitory molecules, which can be explained by the fact that persistent stimulation of T cell receptors provoke PD-1 and Tim-3 production to regulate excessive activation. Consequently, sPD-1, which is produced by the alternative splicing of PD-1 mRNA transcripts, is increased along with enhanced PD-1 production (Fig. 4).

sTim-3 is produced by shedding from the cell surface by a disintegrin and metalloproteinase domain-containing protein (ADAM) 10 or ADAM17 in humans^26,27^. Our results showed that surface Tim-3 was overexpressed in the T cells of patients with high BVAS, which is the source of sTim-3. Consequently, sTim-3 is elevated in patients with active AAV compared with those with inactive AAV. We speculated that activated immunity enhances Tim-3 expression to maintain immune homeostasis, similar to PD-1. However, the immunopathological properties of these soluble molecules in patients with AAV require further investigation.

Not all the molecules showed significant results. For instance, serum sCTLA-4 and sBTLA levels were lower than those of HCs, but decreased further after treatment, and serum sLAG-3 level was higher than those of HCs, but increased further after treatment. Since specific mechanism of production and function of these soluble molecules are little known, further studies are needed to determine how and why these molecules changed in response to treatment.

However, we speculated that chronic immune activation status may influence dysregulation of checkpoint molecules. Dysregulation of soluble immune checkpoint molecules has also been reported in infectious disease other than autoimmune diseases such as hepatitis B virus (HBV), human immunodeficiency virus (HIV), and tuberculosis infection^28–30^. Although detailed function, regulation and mechanism of soluble immune checkpoint molecules in each disease are largely unknown, these findings suggest the possibility that chronic immune activation status may influence dysregulation of checkpoint molecules.

The limitations of this study include the following: first, our data should be interpreted with caution because our results are phenomenological; thus, corroborating mechanistic data are needed to elucidate the underlying mechanism. Downstream signaling following interactions between membrane-bound receptors and soluble checkpoint molecules has not yet been clarified. Second, 15 patients relapsed and were not newly diagnosed; therefore, they were not immunosuppressant-naïve. Therefore, it is possible that the medications affected the soluble checkpoint molecules. Third, all patients were Korean; thus, further studies of various ethnicities are required to validate our results. Fourth, because Miliplex MAP testing is a relatively recently developed technology and we do not have separate validation group reliability and stability test of the Miliplex MAP should be validated in other AAV cohorts in future studies. Nevertheless, the strength of our study is that it is the first to analyze various soluble checkpoint molecules and their associations with AAV-specific indices in a prospective AAV cohort with paired samples.

In conclusion, we demonstrated altered concentrations of serum soluble checkpoint molecules in patients with AAV, as well as a positive correlation between serum sCD28 and sTim-3 concentrations and disease activity. Taken together with our novel findings on the aberrant production of various soluble checkpoint molecules, our results provide new insights into their potential immunopathological roles in AAV and offer novel biomarkers of disease activity in AAV.

## Data Availability

The datasets used and/or analysed during the current study are available from the corresponding author on reasonable request (sangwonleee@yuhs.ac).
